# Cognitive Impairment and Quality of Life in AQP4‐IgG Seropositive Neuromyelitis Optica Spectrum Disorder: A Cross‐Sectional Study in Iranian Patients

**DOI:** 10.1155/nri/5624714

**Published:** 2026-05-09

**Authors:** Saeed Ramazanzadeh, Fereshteh Ashtari

**Affiliations:** ^1^ Neurosciences Research Center, Isfahan University of Medical Sciences, Isfahan, Iran, mui.ac.ir; ^2^ Department of Neurology, Isfahan University of Medical Sciences, Isfahan, Iran, mui.ac.ir

**Keywords:** AQP4-IgG, cognition, MACFIMS, neuromyelitis optica, quality of life

## Abstract

**Background:**

Cognitive impairment (CI) has been reported with wide variability in neuromyelitis optica spectrum disorder (NMOSD), but its impact on patients’ quality of life (QoL) is not well understood. The aim of this study was to describe the cognitive characteristics and various aspects of QoL of NMOSD patients.

**Methods:**

This cross‐sectional study included 49 NMOSD patients (30 seropositive for AQP4‐IgG) and 43 healthy controls (HCs). A set of questionnaires, including information of demographic and clinical characteristics, Multiple Sclerosis Quality of Life‐54 (MSQOL‐54), Beck Depression Inventory (BDI), and Hospital Anxiety and Depression Scale (HADS), was completed. Cognitive assessment was performed using the Minimal Assessment of Cognitive Function in Multiple Sclerosis (MACFIMS) by a trained individual.

**Results:**

Based on CI criteria, 24 patients (48.98%) were classified as cognitively impaired, of whom 20 (83.33%) were AQP4‐IgG seropositive. There was a strong positive correlation between CI and AQP4‐IgG seropositivity (*p* < 0.05). Seropositive patients performed worse on the PASAT (*p* < 0.05) and D‐KEFS (*p* < 0.001) tests of MACFIMS. None of the MSQOL‐54 subscale scores were significantly affected by AQP4‐IgG seropositivity, and there was no significant relationship between the physical or mental health composite score of the MSQOL‐54 and CI.

**Conclusion:**

Our findings suggest that cognitive impairment is a relevant yet underrecognized feature of NMOSD, particularly among AQP4‐IgG seropositive patients. The lack of association between CI and QoL highlights the need for more sensitive, disease‐specific assessment tools. Further longitudinal and multicenter research is warranted to clarify the mechanisms and clinical impact of these impairments.

## 1. Introduction

Neuromyelitis optica spectrum disorder (NMOSD) stands as a serious autoimmune disease affecting the central nervous system. It is characterized by its distinct inclination toward targeting the spinal cord and optic nerves [1], which can lead to debilitating outcomes such as visual impairment and paralysis [2]. Initially subsumed under the spectrum of multiple sclerosis (MS), the discovery of aquaporin‐4 channels protein antibody (AQP4‐IgG) marked a pivotal juncture and facilitated the differentiation between NMOSD and MS [1]. The core clinical presentations of NMOSD include optic neuritis, acute myelitis, area postrema syndrome, acute brainstem syndrome, symptomatic narcolepsy or acute diencephalic clinical syndrome with NMOSD‐typical diencephalic MRI lesions, and symptomatic cerebral syndrome with NMOSD‐typical brain lesions [3].

The prevalence of NMOSD varies widely, depending on the population studied and the diagnostic criteria applied, with estimates ranging from 0.5 to 10 per 100,000 individuals [4]. In Iran, reported prevalence ranges from 0.86 to 1.9 per 100,000 [5, 6]. Although NMOSD is primarily a sporadic disorder, reports of familial clustering suggest a possible interplay between genetic susceptibility and environmental influences, such as dietary habits, lifestyle, history of head trauma, and intentional abortion in women [7]. There is also a strong gender disparity, with women being affected more often than men, this pattern has been observed across diverse ethnic groups, and reported female‐to‐male ratios range from 5:1 to 10:1 [4].

NMOSD patients are at risk of cognitive impairment (CI) [8] that is often underestimated. The prevalence of CI varies across studies, ranging from 3% to 75%, probably due to differences in sample populations, diagnostic criteria, definitions of CI, and the tools used to assess it [1]. Furthermore, these studies provide conflicting views on which cognitive domains are most affected by the disease. In one of our previous studies, NMOSD patients showed poor performances on language, visual perception/spatial processing, and working memory domain tests [9]. A systematic review by Meng et al. found significant impairments in cognitive domains including attention, language, memory, processing speed, and executive function [10].

Several factors can influence CI and its consequences in NMOSD patients, with younger age and higher level of education being associated with better cognitive performance [11]. One of these factors might be AQP4 seropositivity, evidence suggests that AQP4 may play a role in long‐term synaptic plasticity and memory. In animal models, AQP4‐deficient mice demonstrated impaired performance on the Morris water maze, object placement tasks, and contextual fear conditioning, supporting a specific contribution of AQP4 to memory consolidation [12].

Another important consideration in NMOSD patients is the reduced quality of life (QoL) compared to the general population. QoL is a multidimensional construct that reflects an individual’s perception of their position in life within the framework of their cultural and value systems, and in relation to personal goals, expectations, and standards. When applied to health outcomes, this concept is often assessed as health‐related QoL (HRQoL) [13]. Few studies have evaluated the impact of NMOSD on HRQoL systematically [14]; Beekman et al. found that QoL was markedly affected in over 70% of patients, although average emotional well‐being remained relatively preserved [15]. QoL is influenced by multiple factors, including physical health and psychological state, such as pain, anxiety, and depression [14, 16]. Previous studies suggest that depression and anxiety have negative effects on the QoL [17]. In one study, the prevalence of depression in NMOSD patients was reported to be between 42% and 58%, and its severity was associated with decreased scores on information processing tasks [4]. In another study, a lifetime prevalence of depression of 46% was observed in NMOSD patients [18]. Some studies, including Lopez‐Soley et al., have reported that global cognitive performance is linked to the physical impact of NMOSD on QoL [1]. Nevertheless, the relationship between cognitive function and overall QoL in NMOSD patients remains poorly understood. This study aimed to investigate the impact of CI on QoL in NMOSD, as well as the potential influence of AQP4 seropositivity on these outcomes.

## 2. Methods

### 2.1. Participants

This is a cross‐sectional analytical study of NMOSD patients registered at the Kashani MS clinic affiliated with Isfahan University of Medical Sciences, conducted between June 2023 and June 2024. All subjects were definitively diagnosed with NMOSD based on the 2015 international consensus diagnostic criteria [3] and were aged over 18 and under 65 years. Exclusion criteria included (a) acute relapse or corticosteroid therapy within 8 weeks prior to inclusion, (b) history of psychiatric disorders or neurological disorders (apart from NMOSD), (c) severe visual, auditory, or motor impairments that would interfere with the ability to complete the tests, and (d) history of long‐term use of substances or medications that affect cognitive function. Patients who were unwilling to participate could withdraw from the study at any time. The study was approved by the Ethics Committee of Isfahan University of Medical Sciences with the ethical approval code IR.MUI.MED.REC.1402.317, and written informed consent was obtained from all participants at the start of the study. Due to the relative rarity of NMOSD, all NMOSD patients registered at Kashani Hospital were included in the study using a census sampling method, resulting in a final sample size of 49 patients. Furthermore, 43 healthy controls (HC) who were age‐ and sex‐matched to the NMOSD group were selected.

### 2.2. Data Collection Method

Patient data such as age, gender, disease onset age, and education level were extracted from medical records. Disease severity was quantified using the Expanded Disability Status Scale (EDSS) [19]. A trained investigator assisted participants in completing all questionnaires.

### 2.3. Clinical and Cognitive Assessments

The AQP4‐IgG seropositivity was tested using a cell‐based assay. To assess the cognitive status, the Persian‐translated version of the Minimal Assessment of Cognitive Function in Multiple Sclerosis (MACFIMS) battery of neuropsychological tests was used [20]. All neuropsychological assessments, including the MACFIMS battery, were administered in a standardized order by trained examiners in a quiet environment, with two 15‐min breaks provided with refreshments, and participants who experienced fatigue or weakness were allowed to complete the remaining tests on a subsequent day to ensure comfort and data integrity. MACFIMS consists of a series of tests designed to evaluate cognitive status across five domains: processing speed/working memory, learning and memory, executive functions, visual perception/spatial processing, and language/other. The assessment includes the following seven tests: *(a) Paced Auditory Serial Addition Task (PASAT)* measures auditory processing speed, dual‐task attention, and working memory. *(b) Symbol Digit Modalities Test (SDMT):* assess visual processing speed and working memory. *(c) California Verbal Learning Test-Second Edition (CVLT-II):* evaluate verbal memory. *(d) Brief Visuospatial Memory Test-Revised (BVMT-R):* assess visual or spatial episodic memory. *(e) Delis–Kaplan Executive Function System (D-KEFS):* measures higher‐order executive functions and consists of nine subtests, including Trail Making, Verbal Fluency, Design Fluency, Color‐Word, Card Sorting, 20 Questions, Word Context, Tower, and Proverb Test. *(f) Judgment of Line Orientation Test (JLO):* assess spatial processing. *(g) Controlled Oral Word Association Test (COWAT):* which assesses verbal fluency [21].

In regard to the CI definition, there is a sort of disharmony among researchers, some using one test, others two, and some requiring three impaired tests for diagnosis. Furthermore, even among these, some have considered a deficit as one standard deviation below the mean, while others have used as much as two standard deviations [4]. We defined CI as poor performance in at least two of the aforementioned domains, which was determined by a score of one and a half standard deviations below the average score of the general population in corresponding tests, as used in some other studies for a more robust and specific definition [1, 22].

To assess the HRQoL of participants, the Persian version of the Multiple Sclerosis QoL‐54 (MSQoL‐54) questionnaire was used. This questionnaire, based on the Short Form 36 (SF‐36), with an additional 18 MS‐specific items, consists of 52 questions divided into 12 domains (physical health, role limitations due to physical problems, role limitations due to emotional problems, pain, emotional well‐being, energy, health perception, social functioning, cognitive functioning, health distress, overall QoL, and sexual functioning) and two single items (change in health and satisfaction with sexual function). The final score includes 14 scales and two summary scores for physical health and mental health. The composite scores can be calculated by converting item scores to a scale of zero to 100, where zero represents the worst health status and 100 indicating the best health status [23]. The validity and reliability of the Persian version of this questionnaire were calculated by Ghaem and colleagues [24].

Depression was assessed using the Persian version of the Beck Depression Inventory second edition (BDI‐II), a widely used self‐report tool for depression screening in the general population [25]. Anxiety and depression were measured using the Persian version of the Hospital Anxiety and Depression Scale (HADS), whose validity and reliability were confirmed by Montazeri and colleagues [26]. A score ≥ 8 on either subscale of HADS is considered a threshold for clinically relevant anxiety or depression.

### 2.4. Data Analysis Method

After collecting the medical records and completing the questionnaires, the data were entered into SPSS version 27. Normality of continuous variables was assessed using the Shapiro–Wilk test, with *p* values < 0.05 indicating non‐normal distributions. The EDSS score was the only variable that deviated from normality; therefore, Spearman’s rank correlation and the Mann–Whitney *U* test were applied in analyses involving this variable. For normally distributed continuous variables, Pearson correlation coefficients were used. When correlations involved one binary and one continuous variable, point‐biserial correlations were calculated. Homogeneity of variances was assessed using Levene’s test. Independent samples *t*‐tests and chi‐square tests were applied to normally distributed data, while the Mann–Whitney *U* test was used for non‐normally distributed variables, namely the EDSS score. Age, years of education, depression, and anxiety were considered potential confounding variables because of their well‐established associations with cognitive performance. Increasing age and lower educational attainment are known to be related to poorer cognitive function [11], while depressive and anxiety symptoms may adversely affect attention, memory, and executive functioning. To adjust for the potential effects of these variables, the binary logistic regression analysis was performed using SPSS version 27.

### 2.5. Use of Artificial Intelligence Generated Content

The authors used ChatGPT (GPT‐5, OpenAI) to assist in refining the language and checking grammar throughout the manuscript. The tool was employed as a companion to the writing process to improve clarity and readability, without generating or altering the scientific content of the work.

## 3. Results

### 3.1. Demographic, Clinical, and Cognitive Characteristics

The study population consisted of 49 NMOSD patients, of whom 40 (81.6%) were female, and 30 (61.2%) were seropositive for AQP4‐IgG. The demographic and clinical characteristics of NMOSD patients are presented in Table 1.

**TABLE 1:** Demographic and clinical characteristics of neuromyelitis optica spectrum disorder (NMOSD) patients (*N* = 49).

	**Mean (SD)**

Age (y)	40.24 (9.07)
Sex (Male/Female)	9/40 (18.4%/81.6%)
Years of Education (y)	13.02 (4.10)
AQP4‐IgG Status (+/−)	30/19 (61.2%/38.8%)
Age of Onset (y)	27.39 (8.98)
Duration of Disease (y)	12.86 (4.83)
EDSS Score	2.04 (1.16)
Total Number of Attacks	4.38 (2.31)
Number of Attacks in the Past Year	0.74 (0.53)

*Note:* AQP4‐IgG = aquaporin‐4 channels protein antibody.

Abbreviations: EDSS, Expanded Disability Status Scale; SD, standard deviation.

### 3.2. QoL, Depression, and Cognition

In the QoL survey, the mean of physical health composite score was 62.09 (SD 21.39) and for mental health composite was 61.57 (SD 23.11). Depression was assessed using both the BDI and HADS scales, with a mean BDI score of 4.55 (SD = 4.30) in NMOSD and 2.15 in HCs (*p* = 0.02). The mean depression component score on the HADS was 5.66 (SD = 3.88), with 22.4% of scores were equal to or higher than a score of 8, and the mean HADS anxiety component score was 5.59 (SD = 4.66) with 24.5% of scores equal to or above a score of 8.

In the assessment of cognitive function, there was a significant difference between the NMOSD and HCs groups in the PASAT (*p* = 0.001), SDMT (*p* < 0.001), and COWAT (*p* < 0.001) tests. Based on the criteria for CI described earlier, 24 NMOSD patients (48.98%) were classified as cognitively impaired, of which 83.33% (20 patients) were AQP4‐IgG seropositive, or equivalently two‐third of seropositive patients were cognitively impaired. The frequency of impairments in each specific test in the NMOSD group was as follows: 17 patients in the PASAT, 15 patients in the SDMT, 15 patients in the CVLT, 19 patients in the BVMT‐R, 8 patients in the JLO, 16 patients in the COWAT, and 9 patients in the D‐KEFS. Overall, 33 participants showed impairment in at least one cognitive domain, of which 66.66% (22 patients) were AQP4‐IgG seropositive. The clinical and cognitive characteristics of the study participants are presented in Table 2.

**TABLE 2:** Clinical and cognitive characteristics of neuromyelitis optica spectrum disorder (NMOSD) patients and healthy controls (HCs).

	**NMOSD mean (SD)**	**Control mean (SD)**	**p** **value**	**Cohen’s d**

BDI	4.55 (4.30)	2.15 (2.03)^a^	**0.020**	0.633
PASAT	41.06 (13.46)	48.81 (8.66)	**0.001**	−0.676
SDMT	45.59 (14.37)	58.72 (11.27)	**< 0.001**	−1.009
CVLT‐II				
Total	52.39 (11.22)	54.60 (8.49)^a^	0.431	−0.210
SDFR	10.78 (3.24)	12.25 (2.20)^a^	0.066	−0.495
SDCR	11.02 (2.74)	12.65 (2.01)^a^	**0.019**	−0.639
LDFR	10.73 (2.88)	12.30 (2.00)^a^	**0.013**	−0.589
LDCR	11.29 (2.89)	13.35 (2.03)^a^	**0.005**	−0.772
BVMT‐R				
Total	21.33 (9.66)	25.80 (7.40)^a^	**0.044**	−0.493
Delayed recall	9.45 (3.44)	10.40 (2.19)^a^	0.176	−0.303
JLO	19.80 (6.22)	20.90 (5.02)^a^	0.483	−0.187
COWAT	26.47 (10.27)	37.33 (9.70)	**< 0.001**	−1.084
D‐KEFS (D)	22.78 (8.98)	27.00 (8.94)^a^	0.080	−0.471
D‐KEFS (S)	7.12 (2.55)	8.20 (2.42)^a^	0.111	−0.429

*Note:* D = description score; S = total sorting score. Bold values represent *p* values less than 0.05.

Abbreviations: BDI, Beck’s Depression Inventory; BVMT‐R, Brief Visuospatial Memory Test–Revised; COWAT, Controlled Oral Word Association Test; CVLT, California Verbal Learning Test; DKEFS, Delis–Kaplan Executive Function System; HADS, The Hospital Anxiety and Depression Scale; JLO, Judgment of Line Orientation; LDCR, long delayed cued recall; LDFR, long delayed free recall; MSQOL‐54, Multiple Sclerosis Quality of Life‐54; PASAT, Paced Auditory Serial Addition Test; SD, standard deviation; SDCR, short delayed cued recall; SDFR, short delayed free recall; SDMT, Symbol Digit Modalities Test.

^a^For the indicated tests, 23 of the 43 healthy control participants had missing data, whereas all other assessments were completed by all controls.

Characteristics of NMOSD patients, grouped by AQP4 status, are presented in Table 3. In seropositive patients, there was an overall decrease in cognitive test performance compared to seronegative patients, but only PASAT (*p* < 0.05) and D‐KEFS (*p* < 0.001) were significantly reduced, indicating greater impairment in auditory processing speed, working memory, and executive function in this group.

**TABLE 3:** Characteristics of neuromyelitis optica patients grouped by AQP4‐IgG seropositivity.

	**Mean of AQP4- (SD)**	**Mean of AQP4+ (SD)**	**p** **value**	**Cohen’s d**

Age	**36.68 (6.64)**	**42.5 (9.75)**	**0.027**	**−0.669**
Sex (Male/Female)	4/15	5/25	0.706	0.111
EDSS score	2.16 (1.21)	1.96 (1.14)	0.575^a^	
Age of Onset	24.42 (7.97)	29.27 (9.19)	0.065	−0.554
MSQOL‐54 Physical Health	63.89 (20.56)	60.44 (23.29)	0.994	−0.002
MSQOL‐54 Mental Health	59.97 (19.76)	62.91 (25.77)	0.409	−0.244
HADS (Depression)	6.07 (3.52)	5.42 (4.13)	0.615	0.164
HADS (Anxiety)	5.60 (4.64)	5.58 (4.77)	0.988	0.005
BDI	3.87 (4.00)	5.00 (4.73)	0.481	−0.208
PASAT	**48.60 (10.13)**	**37.88 (12.63)**	**0.033**	**0.643**
SDMT	50.67 (13.17)	41.58 (14.84)	0.092	0.505
CVLT‐II				
Total	53.87 (11.84)	51.00 (11.05)	0.270	0.327
SDFR	11.58 (2.76)	10.27 (3.44)	0.169	0.410
SDCR	11.68 (2.43)	10.60 (2.87)	0.179	0.400
LDFR	11.47 (2.78)	10.27 (2.89)	0.155	0.424
LDCR	11.63 (3.58)	11.07 (2.39)	0.510	0.195
BVMT‐R				
Total	24.20 (9.28)	20.12 (9.74)	0.064	0.556
Delayed Recall	10.00 (3.42)	9.15 (3.54)	0.255	0.338
JLO	20.73 (6.71)	18.73 (5.97)	0.265	0.331
COWAT	28.76 (9.57)	25.35 (10.82)	0.396	0.251
D‐KEFS (D)	**27.93 (10.19)**	**18.35 (6.22)**	**0.001**	**1.253**
D‐KEFS (S)	**8.53 (2.95)**	**6.00 (1.92)**	**0.001**	**1.089**

*Note:* AQP4‐IgG = aquaporin‐4 channels protein antibody; D = description score; S = total sorting score. Bold values represent *p* values less than 0.05.

Abbreviations: BDI, Beck’s Depression Inventory; BVMT‐R, Brief Visuospatial Memory Test–Revised; COWAT, Controlled Oral Word Association Test; CVLT, California Verbal Learning Test; DKEFS, Delis–Kaplan Executive Function System; HADS, The Hospital Anxiety and Depression Scale; JLO, Judgment of Line Orientation; LDCR, long delayed cued recall; LDFR, long delayed free recall; MSQOL‐54, Multiple Sclerosis Quality of Life‐54; PASAT, Paced Auditory Serial Addition Test; SD, standard deviation; SDCR, short delayed cued recall; SDFR, short delayed free recall; SDMT, Symbol Digit Modalities Test.

^a^Mann–Whitney’s asymptotic significance (2‐tailed).

There were no other significant differences between AQP4‐IgG seropositive and seronegative patient groups in regard to the age of onset of disease (*p* = 0.065), EDSS score (*p* = 0.575), sex (*p* = 0.706), and QoL. As shown in Table 4, none of the MSQOL‐54 subscale scores were significantly affected by the AQP4‐IgG seropositivity.

**TABLE 4:** MSQOL‐54 scale scores grouped by AQP4‐IgG seropositivity.

	**Mean (SD)**	**Mean of AQP4- (SD)**	**Mean of AQP4+ (SD)**	**p** **value**

Physical Health	53.30 (30.63)	57.10 (30.29)	50.90 (31.11)	0.495
Role Limitation due to Physical Problems	60.37 (43.15)	55.26 (41.31)	63.61 (44.66)	0.515
Role Limitation due to Emotional Problems	56.46 (48.21)	50.88 (48.90)	60.00 (48.26)	0.524
Pain	68.52 (25.49)	65.96 (26.58)	70.14 (24.94)	0.581
Emotional Well‐Being	54.27 (19.02)	49.68 (15.74)	57.17 (20.56)	0.183
Energy	48.22 (8.43)	46.94 (7.28)	49.02 (9.10)	0.407
Health Perception	62.02 (21.16)	65.79 (17.58)	59.63 (23.11)	0.326
Social Function	75.68 (22.75)	72.81 (25.13)	77.50 (21.35)	0.487
Cognitive Function	71.94 (26.65)	63.95 (32.68)	77.00 (21.07)	0.133
Health Distress	68.67 (25.16)	71.58 (18.93)	66.83 (28.57)	0.488
Sexual Function	66.67 (38.29)	67.86 (37.71)	65.97 (40.27)	0.921
Change in Health	61.22 (28.90)	50.00 (27.64)	68.33 (27.80)	0.029
Satisfaction with Sexual Function	59.62 (31.73)	50.00 (39.53)	67.05 (22.34)	0.125
Overall Quality of Life	66.49 (19.71)	65.96 (18.68)	66.84 (20.67)	0.882

*Note:* AQP4‐IgG = aquaporin‐4 channels protein antibody.

Abbreviations: MSQOL‐54, Multiple Sclerosis Quality of Life‐54; SD, standard deviation.

We found a strong positive correlation between CI and serum AQP4‐IgG positivity (Phi coefficient = 0.445, *p* < 0.05), depression, age, and EDSS score, as well as a moderate negative correlation between CI and years of education (Spearman correlation coefficient = −0.454, *p* < 0.05). QoL was compared in patients with and without CI, and no statistically significant correlation was observed between the physical health composite score (*p* = 0.157) or the mental health composite score (*p* = 0.236) and CI. As shown in Table 5, CI was not significantly associated with either anxiety or disease duration.

**TABLE 5:** Correlations between cognitive impairment^a^ and clinical and demographic characteristics of NMOSD patients.

	**Correlation coefficient**	**p** **value**

HADS (Anxiety)	0.116^b^	0.469
HADS (Depression)	**0.342** ^b^	**0.029**
BDI	**0.318** ^b^	**0.026**
MSQOL‐54 Physical Health	−0.205	0.157
MSQOL‐54 Mental Health	−0.172	0.236
AQP4‐IgG Status	**0.445** ^c^	**0.002**
Age	**0.455** ^d^	**0.001**
Years of Education	**−0.410** ^b^	**0.003**
Duration of Disease	0.166	0.254
EDSS Score	**0.307** ^b^	**0.036**

*Note:* NMOSD = Neuromyelitis Optica Spectrum Disorder; AQP4‐IgG = aquaporin‐4 channels protein antibody.

Abbreviations: BDI, Beck’s Depression Inventory; EDSS, Expanded Disability Status Scale; HADS, The Hospital Anxiety and Depression Scale; MSQOL‐54, Multiple Sclerosis Quality of Life‐54.

^a^Defined by a score of one and a half standard deviations below the average score of the control group in at least two cognitive domains.

^b^Spearman’s correlation coefficient is reported due to not satisfying the normal distribution condition.

^c^Phi coefficient is reported for these two binary variables.

^d^Equality of variances was not reached by using Levene’s test for equality of variances (*p* = 0.048). Spearman’s correlation coefficient is reported.

Furthermore, we observed a low positive correlation between age and AQP4‐IgG seropositivity (correlation coefficient = 0.316, *p* = 0.027). Age was considered a confounding variable in the correlation between CI and AQP4‐IgG seropositivity. After adjusting for this confounder, the correlation between CI and AQP4‐IgG seropositivity remained statistically significant (*p* = 0.022). When we considered years of education as another confounding variable, the correlation between CI and AQP4‐IgG seropositivity remained significant (*p* = 0.033). We also considered other confounders such as disability and depression as shown in Table 6.

**TABLE 6:** Correlation between cognitive impairment and AQP4‐IgG seropositivity in NMOSD patients, adjusted for potential confounding factors.

	**Odd ratio**	**p** **value**

AQP4‐IgG Status	**8.51**	**0.025**
Age	1.09	0.106
Years of Education	0.83	0.138
EDSS Score	**2.69**	**0.026**
BDI	1.13	0.239

*Note:* NMOSD = Neuromyelitis Optica Spectrum Disorder; AQP4‐IgG = aquaporin‐4 channels protein antibody. Bold values represent *p* values less than 0.05.

Abbreviations: BDI, Beck’s Depression Inventory; EDSS, Expanded Disability Status Scale.

## 4. Discussion

This study primarily aimed to evaluate cognitive function and QoL in NMOSD patients and to determine whether these outcomes differ by AQP4‐IgG seropositivity. To our knowledge, it is the first investigation to examine the relationship between cognitive impairment, QoL, and AQP4‐IgG seropositivity in an Iranian NMOSD population.

Due to the importance of CI and its prevalence in NMOSD patients, it seems necessary to investigate the properties of cognitive performance in this population. The wide variability in reported CI prevalence underscores the need for a standardized, NMOSD‐specific cognitive assessment battery [1]. In our study, 48.98% of NMOSD patients had CI, of whom 83.33% were AQP4‐IgG seropositive. Seropositive patients showed overall poorer cognitive performance than seronegative patients, particularly in auditory processing speed, working memory, and executive function. A strong positive association between CI and AQP4‐IgG seropositivity remained significant after adjusting for confounders including age, depression, anxiety, disability, and education. These findings contrast with previous research, which found no significant differences between seropositive and seronegative NMOSD patients [27]. Our results may relate to the role of AQP4 in synaptic plasticity [12, 28] and the influence of AQP4 on cognitive functions and memory [29]. Inhibition of AQP4 leads to increased amyloid deposition and deficient behavior. Proper polarization of AQP4 in astrocytes is essential for the effective functioning of the brain’s glymphatic system. It has been suggested that proper polarization of AQP4 in astrocytes may be important for maintaining the efficiency of the brain’s glymphatic system, which contributes to metabolic waste clearance [30]. Experimental findings also indicate that inhibition or dysregulation of AQP4 could be associated with impaired glymphatic function, increased amyloid deposition, and cognitive alterations [31].

NMOSD patients in our sample performed worse than HCs in processing speed, working memory, and verbal fluency. Similarly, Blanc et al. reported significant deficits in attention, processing speed, and verbal fluency among NMOSD patients compared with controls [32]. Our findings also align with prior studies showing that cognitive performance in NMOSD is influenced by age and education, with lower education identified as a predictor of CI [4, 33].

In contrast to a study that showed lower QoL in seronegative NMOSD patients [2], we did not observe any statistically significant association between AQP4‐IgG seropositivity and QoL in our study. Similarly, no association was found between CI and QoL, and mean MSQOL‐54 scores did not differ significantly between patients with and without CI. These discrepancies may be explained by the modest sample size of our study, the absence of NMOSD‐specific tools for assessing cognition and QoL, and the lack of consensus regarding the definition of CI in this population.

Our data show a moderate correlation between EDSS scores and CI, but we did not find a significant difference between EDSS score and seropositivity. In line with our findings, there are studies that show an association between higher EDSS score and CI [18, 34]. On the other hand, there are studies that claim that CI does not necessarily occur in patients with severe physical dysfunction [4].

Previous studies have shown that depression is more frequent and severe in NMOSD patients compared to the general population [33], affecting approximately half of patients with NMOSD. Higher severity of depressive symptoms was associated with more pronounced CI [4], suggesting a close relationship between psychological well‐being and CI [1]. In line with the general trend reported in previous studies, our findings also showed higher depression scores in NMOSD patients compared to HCs. Moreover, we observed a moderate positive correlation between CI and depression, whereas no significant association was found between CI and anxiety. Also, depression and anxiety scores did not differ between seropositive and negative groups. Similar to other reports, we did not observe a relationship between the duration of NMOSD disease and cognitive test performance [1, 18, 33].

## 5. Implication and Usage

By elucidating the cognitive impacts of NMOSD and the role of AQP4‐IgG seropositivity, this research could inform researchers and clinicians about the necessity of comprehensive assessments and unified batteries of tests that include cognitive evaluations, ultimately improving patient care.

## 6. Limitations

There were some limitations regarding this study. Firstly, its cross‐sectional analytical design and lack of follow‐up for exploring causality or dynamic relations. Secondly, the study was conducted in one referral medical center and has a small sample size. Thirdly, the absence of a standardized definition for cognitive CI in the scientific literature, coupled with the lack of specific cognitive test batteries tailored for NMOSD, creates inconsistencies that hinder the comparability of results across studies. A further limitation of this study was the presence of missing data in the HC group. Complete assessment data were available for only 20 of the 43 controls, while 23 participants had incomplete test results across several cognitive and psychological measures. This incomplete data collection may have reduced statistical power and should be considered when interpreting comparisons between patients and HCs.

## 7. Conclusion

In our study, 48.98% of patients with NMOSD had CI, and there was a strong correlation between AQP4‐IgG positivity and CI. Processing speed, verbal memory, working memory, and executive functions were the most affected cognitive domains. There was no significant association between CI and HRQoL in NMOSD patients. These findings may be partly explained by the modest sample size, the lack of NMOSD‐specific tools for assessing cognition and QoL, and the absence of a standardized definition of CI in this population. Clinically, our results emphasize that cognitive dysfunction, particularly among AQP4‐IgG seropositive patients, warrants attention and routine assessment. Future studies should employ longitudinal, multicenter designs with larger sample sizes and standardized, NMOSD‐specific tools for assessing cognition and QoL to better elucidate these associations. Incorporating genetic, environmental, and neuroimaging factors is also recommended to achieve a more comprehensive understanding. Additionally, interventional studies aimed at developing targeted strategies for managing CI in NMOSD would be valuable.

## Author Contributions

Saeed Ramazanzadeh performed the investigation, data curation, formal analysis, and drafted the original manuscript. Fereshteh Ashtari contributed to the study conceptualization and methodology, supervised the project, and reviewed and edited the manuscript.

## Funding

This study was financially supported by the Isfahan University of Medical Sciences, which facilitated the completion of this study.

## Disclosure

This article is based on the MD thesis of Saeed Ramazanzadeh, defended at Isfahan University of Medical Sciences in 2025 [35].

## Conflicts of Interest

The authors declare no conflicts of interest.

## Data Availability

The data that support the findings of this study are available from the corresponding author upon reasonable request.

## References

[1] Lopez-Soley E. , Meca-Lallana J. E. , Llufriu S. et al., Cognitive Performance and Health-Related Quality of Life in Patients With Neuromyelitis Optica Spectrum Disorder, Journal of Personalized Medicine. (May 2022) 12, no. 5, 10.3390/jpm12050743. PMC9146457 35629165

[2] Holroyd K. B. , Manzano G. S. , and Levy M. , Update on Neuromyelitis Optica Spectrum Disorder, Current opinion in ophthalmology. NLM (Medline). (2020) 31, no. 6, 462–468, 10.1097/ICU.0000000000000703. PMC7771018 33009077

[3] Wingerchuk D. M. , Banwell B. , Bennett J. L. et al., International Consensus Diagnostic Criteria for Neuromyelitis Optica Spectrum Disorders, Neurology. (July 2015) 85, no. 2, 177–189, 10.1212/wnl.0000000000001729, 2-s2.0-84940730364. 26092914 PMC4515040

[4] Czarnecka D. , Oset M. , Karlińska I. , and Stasiołek M. , Cognitive Impairment in NMOSD—More Questions than Answers, Brain and Behavior. (2020) 10, no. 11, 10.1002/brb3.1842. PMC7667314 33022898

[5] Rezaeimanesh N. , Sahraian M. A. , Moghadasi A. N. , and Eskandarieh S. , Epidemiology of Neuromyelitis Optica Spectrum Disorder in Tehran, Iran: the Prevalence, Baseline Characteristics, and Clinical Aspects, Neurological Sciences. (2020) 41, no. 9, 2647–2648, 10.1007/s10072-020-04393-7. 32306139

[6] Papp V. , Magyari M. , Aktas O. et al., Worldwide Incidence and Prevalence of Neuromyelitis Optica: A Systematic Review, Neurology. (January 2021) 96, no. 2, 59–77, 10.1212/wnl.0000000000011153. 33310876 PMC7905781

[7] Eskandarieh S. , Nedjat S. , Abdollahpour I. et al., Environmental Risk Factors in Neuromyelitis Optica Spectrum Disorder: A Case–Control Study, Acta Neurologica Belgica. (June 2018) 118, no. 2, 277–287, 10.1007/s13760-018-0900-5, 2-s2.0-85045142827. 29500680

[8] Ashtari F. , Najarzadeh P. , Shaygannejad V. et al., Cognitive Function and Brain Magnetic Resonance Imaging Profiles in Neuromyelitis Optica Spectrum Disorder and Multiple Sclerosis, Journal of Research in Medical Sciences. (August 2024) 29, no. 1, 10.4103/jrms.jrms_703_23. PMC11472874 39403229

[9] Barzegar M. , Ashtari F. , Kafieh R. et al., Association of Peripapillary Retinal Nerve Fiber Layer Atrophy with Cognitive Impairment in Patients With Neuromyelitis Optica Spectrum Disorder, Neurological Sciences. (2024) 46. 10.1007/s10072-024-07897-8 39609354

[10] Meng H. , Xu J. , Pan C. et al., Cognitive Dysfunction in Adult Patients With Neuromyelitis Optica: A Systematic Review and Meta-Analysis, Journal of Neurology. Dr. Dietrich Steinkopff Verlag GmbH and Co. KG. (2017) 264, no. 8, 1549–1558, 10.1007/s00415-016-8345-3, 2-s2.0-85001124694. 27909800

[11] Moghadasi A. N. , Mirmosayyeb O. , Mohammadi A. , Sahraian M. A. , and Ghajarzadeh M. , The Prevalence of Cognitive Impairment in Patients With Neuromyelitis Optica Spectrum Disorders (NMOSD): A Systematic Review and Meta-Analysis, Multiple Sclerosis and Related Disorders. (2021) 49, 10.1016/j.msard.2021.102757. 33486400

[12] Szu J. I. and Binder D. K. , The Role of Astrocytic Aquaporin-4 in Synaptic Plasticity and Learning and Memory, Frontiers in Integrative Neuroscience. (2016) 10, 10.3389/fnint.2016.00008, 2-s2.0-84961746355. PMC4764708 26941623

[13] Pequeno N. P. F. , Pequeno N. P. F. , Cabral N. L. de A. , Marchioni D. M. , Lima S. C. V. C. , and Lyra C. de O. , Quality of Life Assessment Instruments for Adults: A Systematic Review of Population-based Studies, Health and Quality of Life Outcomes. BioMed Central. (2020) 18, no. 1, 10.1186/s12955-020-01347-7. PMC7329518 32605649

[14] Carnero Contentti E. , Eizaguirre M. B. , López P. A. , Rojas J. I. , Tkachuk V. , and Alonso R. , Health-Related Quality of Life in Neuromyelitis Optica Spectrum Disorder Patients in an Argentinean Cohort, Multiple Sclerosis and Related Disorders. (March 2022) 59. 10.1016/j.msard.2022.103647 35124305

[15] Beekman J. , Keisler A. , Pedraza O. et al., Neuromyelitis Optica Spectrum Disorder: Patient Experience and Quality of Life, Neurol Neuroimmunol Neuroinflamm. (July 2019) 6, no. 4, 10.1212/nxi.0000000000000580, 2-s2.0-85073761663. PMC6624099 31355316

[16] Kim S. , Lee E. J. , Kim K. W. et al., Quality of Life of Patients With Multiple Sclerosis and Neuromyelitis Optica Spectrum Disorders: Cross-Sectional and Longitudinal Analysis, Multiple Sclerosis and Related Disorders. (February 2022) 58, 10.1016/j.msard.2022.103500. 35032884

[17] Nakazawa K. , Noda T. , Ichikura K. et al., Resilience and Depression/Anxiety Symptoms in Multiple Sclerosis and Neuromyelitis Optica Spectrum Disorder, Multiple Sclerosis and Related Disorders. (2018) 25, 309–315, 10.1016/j.msard.2018.08.023, 2-s2.0-85052516987. 30176401

[18] Moore P. , Methley A. , Pollard C. et al., Cognitive and Psychiatric Comorbidities in Neuromyelitis Optica, Journal of the Neurological Sciences. (January 2016) 360, 4–9, 10.1016/j.jns.2015.11.031, 2-s2.0-84955506417. 26723962

[19] Kurtzke J. F. , Rating Neurologic Impairment in Multiple Sclerosis: An Expanded Disability Status Scale (EDSS), Neurology. (November 1983) 33, no. 11, 10.1212/wnl.33.11.1444. 6685237

[20] Eshaghi A. , Riyahi-Alam S. , Roostaei T. et al., Validity and Reliability of a Persian Translation of the Minimal Assessment of Cognitive Function in Multiple Sclerosis (MACFIMS), Clinical Neuropsychologist. (August 2012) 26, no. 6, 975–984, 10.1080/13854046.2012.694912, 2-s2.0-84871866800. 22681459

[21] Benedict R. H. B. , Cookfair D. , Gavett R. et al., Validity of the Minimal Assessment of Cognitive Function in Multiple Sclerosis (MACFIMS), Journal of the International Neuropsychological Society. (2006) 37. 10.1017/s1355617706060723 16981607

[22] Liu Y. , Fu Y. , Schoonheim M. M. et al., Structural MRI Substrates of Cognitive Impairment in Neuromyelitis Optica, Neurology. (October 2015) 85, no. 17, 1491–1499, 10.1212/wnl.0000000000002067, 2-s2.0-84945282616. 26423432

[23] Huang W. , ZhangBao J. , Chang X. et al., Neuromyelitis Optica Spectrum Disorder in China: Quality of Life and Medical Care Experience, Multiple Sclerosis and Related Disorders. (November 2020) 46, 10.1016/j.msard.2020.102542. 33296965

[24] Ghaem H. , Ab H. , Jafari P. , and Nikseresht A. , Validity and Reliability of the Persian Version of the Multiple Sclerosis Quality of Life Questionnaire, Neurology India. (2007) 55, no. 4, 10.4103/0028-3886.33316, 2-s2.0-36549073144. 18040110

[25] Ghassemzadeh H. , Mojtabai R. , Karamghadiri N. , and Ebrahimkhani N. , Psychometric Properties of a Persian-Language Version of the Beck Depression Inventory—Second Edition: BDI-II-PERSIAN, Depression and Anxiety. (2005) 21, no. 4, 185–192, 10.1002/da.20070, 2-s2.0-23844505268. 16075452

[26] Montazeri A. , Vahdaninia M. , Ebrahimi M. , and Jarvandi S. , Health and Quality of Life Outcomes the Hospital Anxiety and Depression Scale (HADS): Translation and Validation Study of the Iranian Version [Internet], 2003, https://www.hqlo.com/content/1/1/14. 10.1186/1477-7525-1-14 PMC161819 12816545

[27] Hümmert M. W. , Stern C. , Paul F. et al., Cognition in Patients With Neuromyelitis Optica Spectrum Disorders: A Prospective Multicentre Study of 217 Patients (CogniNMO-Study), Multiple Sclerosis. (June 2023) 29, no. 7, 819–831, https://pubmed.ncbi.nlm.nih.gov/36786424/. 36786424 10.1177/13524585231151212 PMC10278388

[28] Scharfman H. E. and Binder D. K. , Aquaporin-4 Water Channels and Synaptic Plasticity in the Hippocampus, Neurochemistry International. (December 2013) 63, no. 7, 702–711, https://pubmed.ncbi.nlm.nih.gov/23684954/, 10.1016/j.neuint.2013.05.003, 2-s2.0-84888199965. 23684954 PMC3783552

[29] Hubbard J. A. , Szu J. I. , and Binder D. K. , The Role of Aquaporin-4 in Synaptic Plasticity, Memory and Disease, Brain Res Bull [Internet]. (January 2018) 136, 118–129, https://pubmed.ncbi.nlm.nih.gov/28274814/, 10.1016/j.brainresbull.2017.02.011, 2-s2.0-85014585181. 28274814

[30] Alavian F. and Hajimohammadi A. , Interplay Between Gut Microbiome and Glymphatic System in Cognitive Function and Memory Regulation, Molecular Biology Reports. (December 2025) 52, no. 1, https://pubmed.ncbi.nlm.nih.gov/41099783/, 10.1007/s11033-025-11129-3. 41099783

[31] Manescu M. D. , Catalin B. , Baldea I. et al., Aquaporin 4 Modulation Drives Amyloid Burden and Cognitive Abilities in an APPPS1 Mouse Model of Alzheimer’s Disease, Alzheimers Dement. (May 2025) 21, no. 5, https://pubmed.ncbi.nlm.nih.gov/40329616/, 10.1002/alz.70164. PMC12056304 40329616

[32] Blanc F. , Zéphir H. , Lebrun C. et al., Cognitive Functions in Neuromyelitis Optica, Archives of Neurology. (January 2008) 65, no. 1. 10.1001/archneurol.2007.16 18195143

[33] Hollinger K. R. , Franke C. , Arenivas A. et al., Cognition, Mood, and Purpose in Life in Neuromyelitis Optica Spectrum Disorder, Journal of the Neurological Sciences. (March 2016) 362, 85–90, 10.1016/j.jns.2016.01.010, 2-s2.0-84960863437. 26944124

[34] Ashtari F. , Manouchehri N. , Shaygannejad V. et al., Assessment of Intelligence Quotient in Patients With Neuromyelitis Optica Spectrum Disease and Multiple Sclerosis, Multiple Sclerosis and Related Disorders. (February 2023) 70, 10.1016/j.msard.2022.104492. 36587484

[35] Ramazanzadeh S. and Ashtari F. , Cognitive Impairment and Quality of Life in Iranian Patients With Neuromyelitis Optica Spectrum Disorder: A Cross-Sectional Study, School of Medicine, Isfahan University of Medical Sciences. (2025) https://med.mui.ac.ir/fa/%D9%BE%D8%A7%DB%8C%D8%A7%D9%86-%D9%86%D8%A7%D9%85%D9%87-%D9%87%D8%A7%DB%8C-%DA%A9%D8%A7%D8%B1%D9%88%D8%B1%D8%B2%DB%8C-%D8%AF%D8%A7%D8%AE%D9%84%DB%8C-%D8%A7%D8%B9%D8%B5%D8%A7%D8%A8.

